# Targeting Lysosomes in Colorectal Cancer: Exploring the Anticancer Activity of a New Benzo[*a*]phenoxazine Derivative

**DOI:** 10.3390/ijms24010614

**Published:** 2022-12-29

**Authors:** João C. C. Ferreira, Sara Granja, Ana F. Almeida, Fátima Baltazar, M. Sameiro T. Gonçalves, Ana Preto, Maria João Sousa

**Affiliations:** 1 Centre of Molecular and Environmental Biology (CBMA), Department of Biology, University of Minho, Campus of Gualtar, 4710-057 Braga, Portugal; 2 IBS-Institute of Science and Innovation for Bio-Sustainability, University of Minho, Campus of Gualtar, 4710-057 Braga, Portugal; 3 Centre of Chemistry, Department of Chemistry, University of Minho, Campus of Gualtar, 4710-057 Braga, Portugal; 4 Department of Pathological, Cytological and Thanatological Anatomy, School of Health, Polytechnic Institute of Porto, 4200-072 Porto, Portugal; 5 Life and Health Sciences Research Institute (ICVS), School of Medicine, University of Minho, 4710-057 Braga, Portugal; 6 ICVS/3B’s-PT Government Associate Laboratory, 4710-057 Braga, Portugal; 7 ICVS/3B’s-PT Government Associate Laboratory, 4806-909 Guimarães, Portugal

**Keywords:** Nile Blue analogue, benzo[*a*]phenoxazine, anticancer drug, colorectal cancer, lysosome membrane permeabilization

## Abstract

Colorectal cancer (CRC) has been ranked as one of the cancer types with a higher incidence and one of the most mortal. There are limited therapies available for CRC, which urges the finding of intracellular targets and the discovery of new drugs for innovative therapeutic approaches. In addition to the limited number of effective anticancer agents approved for use in humans, CRC resistance and secondary effects stemming from classical chemotherapy remain a major clinical problem, reinforcing the need for the development of novel drugs. In the recent years, the phenoxazines derivatives, Nile Blue analogues, have been shown to possess anticancer activity, which has created interest in exploring the potential of these compounds as anticancer drugs. In this context, we have synthetized and evaluated the anticancer activity of different benzo[*a*]phenoxazine derivatives for CRC therapy. Our results revealed that one particular compound, BaP1, displayed promising anticancer activity against CRC cells. We found that BaP1 is selective for CRC cells and reduces cell proliferation, cell survival, and cell migration. We observed that the compound is associated with reactive oxygen species (ROS) generation, accumulates in the lysosomes, and leads to lysosomal membrane permeabilization, cytosolic acidification, and apoptotic cell death. *In vivo* results using a chicken embryo choriollantoic membrane (CAM) assay showed that BaP1 inhibits tumor growth, angiogenesis, and tumor proliferation. These observations highlight that BaP1 as a very interesting agent to disturb and counteract the important roles of lysosomes in cancer and suggests BaP1 as a promising candidate to be exploited as new anticancer lysosomal-targeted agent, which uses lysosome membrane permeabilization (LMP) as a therapeutic approach in CRC.

## 1. Introduction

Colorectal cancer (CRC) is reported as the third most diagnosed cancer and the second deadliest worldwide. Despite primary prevention being the key strategy to reduce the impact of CRC, with the growth of the world population associated with poor lifestyle choices, it is expected that the global burden of CRC will increase [1]. Overall, the number of effective CRC chemotherapeutic agents approved for use in humans is still very limited [2]. Moreover, tumor resistance and secondary effects stemming from the classical chemotherapy remain a major clinical problem, reinforcing the need for the identification of new intracellular targets and the discovery of drugs with an effective action [3,4]. In this field, lysosomes have been emerging as attractive targets for the development of new drugs [5,6]. The lysosomes are single membrane-enclosed cytoplasmic organelles and are the main digestive structure in eukaryotic cells, playing critical roles in several cellular processes such as autophagy, apoptosis, protein maturation, membrane repair, cell signaling, and energy metabolism [7,8,9,10]. Lysosome function and dysfunction have been found to play important roles in human disease, including cancer. Cancer cells have numerous, relatively large and acidic lysosomes, and these are thought to be more fragile than normal-sized lysosomes [11,12]. Overexpression of lysosomal proteases is commonly observed in cancer cells, which often correlates with poor prognosis and increased recurrence of many cancers [6,13]. In addition, it has been reported that cancer cell lysosomes are associated with drug resistance through drug sequestration, whereby substances become trapped in the acidic lumen of lysosomes [14,15]. Thus, it is clear that the lysosome arises as a promising therapeutical target as it shows vulnerability that can be exploited through the use of lysosome-targeting agents.

Phenoxazine compounds have been reported mainly for their applications as fluorescent probes, such as is the case of Nile Blue [16,17,18,19,20,21,22]. However, this class of compounds has shown to possess antiproliferative activity, which has increased the interest in evaluating them as pharmaceutical drugs. In fact, there are several reports of different activities for these compounds, such as antifungal [22,23,24,25], antimalarial [26,27], antibacterial [28,29], antiviral [30], and antitumor [31,32]. Still, despite the interest, the information available regarding their mechanisms of action is still limited. As such, in the past years, our group has been designing and synthesizing novel benzo[*a*]phenoxazine compounds and exploring them for potential pharmacological application using yeast and mammalian cells as complementary models.

**Figure 1:**
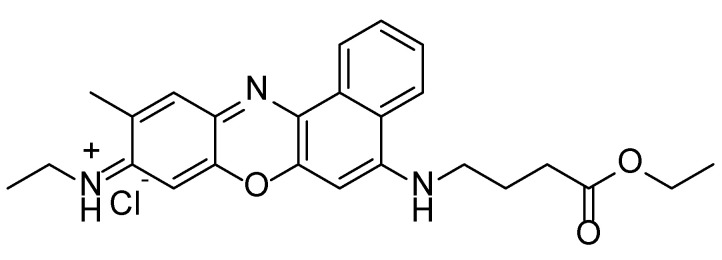
Chemical structure of *N*-(5-((4-ethoxy-4-oxobutyl)amino)-10-methyl-9*H*-benzo[*a*]phenoxazin-9-ylidene)ethanaminium chloride (BaP1).

In our previous work, we used the yeast *Saccharomyces cerevisiae* as a eukaryotic cell model and performed a detailed characterization of the effect of one of our most active compounds, BaP1 (Figure 1). We found that BaP1 accumulates in the vacuole, the yeast lysosome equivalent organelle, and induced a regulated cell death process associated with vacuolar membrane permeabilization [23].

Here, we aimed at further explore BaP1 potential, evaluate its application as an anticancer drug for CRC treatment and understand the underlying mechanisms associated with its activity.

## 2. Results

### 2.1. Colorectal Cancer Cells Are More Sensitive to BaP1 Compared to Noncancerous Colon Cells

**Figure 2:**
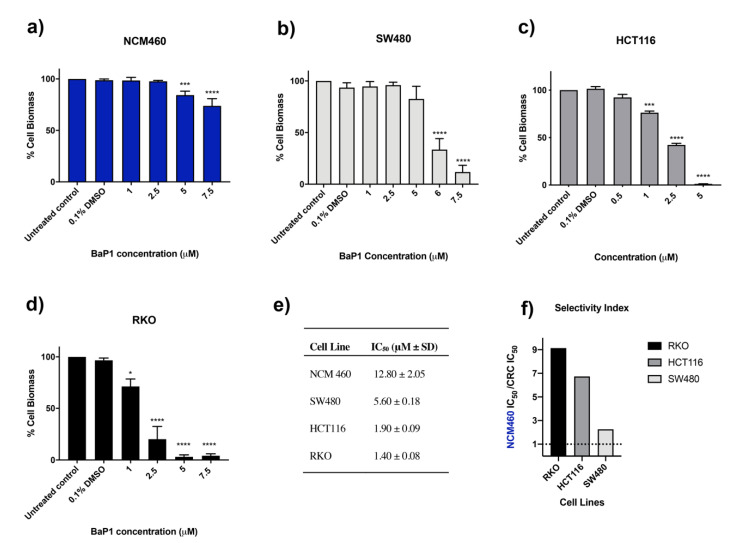
Effect of BaP1 on cell viability of (**a**) NCM460, (**b**) SW480, (**c**) HCT116, and (**d**) RKO cell lines. Cell lines were exposed to increasing concentrations of BaP1 or DMSO (0.1%) for 48 h. After the incubation, cell biomass was assessed by sulforhodamine B (SRB) assay. Values are means with SD (n ≥ 3). Statistical analysis was performed by one-way ANOVA. * *p* < 0.05, *** *p* < 0.001, **** *p* < 0.0001. (**e**) IC_50_ values determined after the respective incubation time. (**f**) *In vitro* selectivity index (NCM460 IC_50_/CRC cell lines IC_50_). A broken line at selectivity index = 1 represents no difference in IC_50_ between tumor and normal cells.

BaP1 activity and selectivity were investigated using the noncancerous colon cell line NCM460 and CRC-derived cell lines harboring somatic mutations on KRAS or BRAF, SW480 (KRAS^G12V^), HCT116 (KRAS^G13D^), and RKO (BRAF^V600E^). Exposure to increasing concentrations of BaP1 decreased the viability of the cell lines, with different sensitivities (Figure 2). The noncancerous colon derived cell line NCM460 exhibited the highest resistance to the drug, with an IC_50_ of 12.8 μM (Figure 2a,e). CRC cells were more sensitive to the effect of BaP1; SW480 and HCT116 exhibited IC_50_ values of 5.6 μM and 1.9 μM, and selectivity indices of 2.26 and 6.7, respectively (Figure 2b,c,e,f). The RKO cell line was the most sensitive showing significant inhibition of cell growth at low doses of the drug, with a resultant IC_50_ of 1.4 μM and a high selectivity index of over 9 (Figure 2d–f).

### 2.2. BaP1 Inhibits Proliferation, Reduces Cell Survival and Motility, and Leads to ROS Generation in RKO Cells

**Figure 3:**
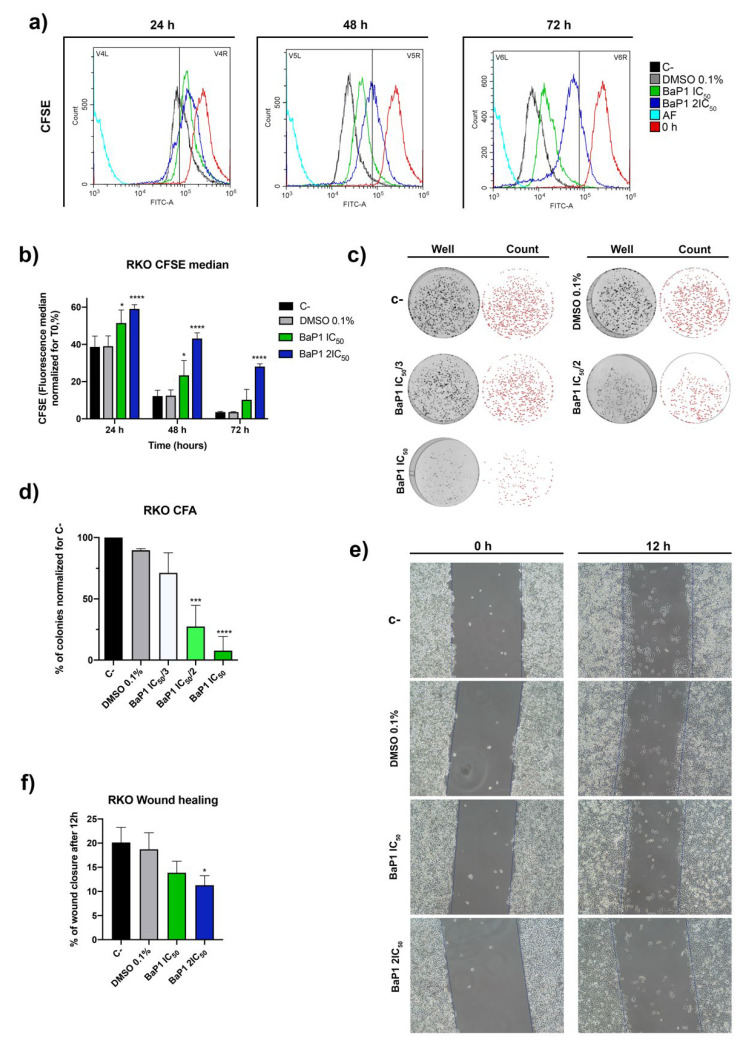
BaP1 biological effects on RKO cell line. (**a**) Representative histograms of RKO cell proliferation analysis with Carboxyfluorescein Diacetate Succinimidyl Ester (CFSE) after 0, 24, 48, and 72 h of exposure to BaP1 IC_50_ and 2 × IC_50_. Untreated cells (C-) and DMSO (0.1%) exposed cells were used as negative controls. (**b**) Quantification of the CFSE fluorescence median; values normalized to T0 after 24, 48, and 72 h of exposure. Values are means with SD (n ≥ 3). Statistical analysis was performed by two-way ANOVA. * *p* < 0.05, **** *p* < 0.0001. (**c**) Representative images of the Colony Formation Assay after exposure to increasing concentrations of BaP1 (IC_50_/3, IC_50_/2, and IC_50_) for 48 h. Untreated cells (C-) and DMSO (0.1%) exposed cells were used as negative controls. (**d**) Quantification of the number of colonies; values normalized to C-. Values are means with SD (n ≥ 3). Statistical analysis was performed by one-way ANOVA. *** *p* < 0.001, **** *p* < 0.0001. (**e**) Representative images of wound healing migration assay after 0 and12 h of exposure to BaP1 IC_50_ and 2×IC_50_. Untreated cells (C-) and DMSO (0.1%) exposed cells were used as negative controls. (**f**) Analysis of the % of wound closure after 12 h. Values are means with SD (n ≥ 3). Statistical analysis was performed by one-way ANOVA. * *p* < 0.05.

**Figure 4:**
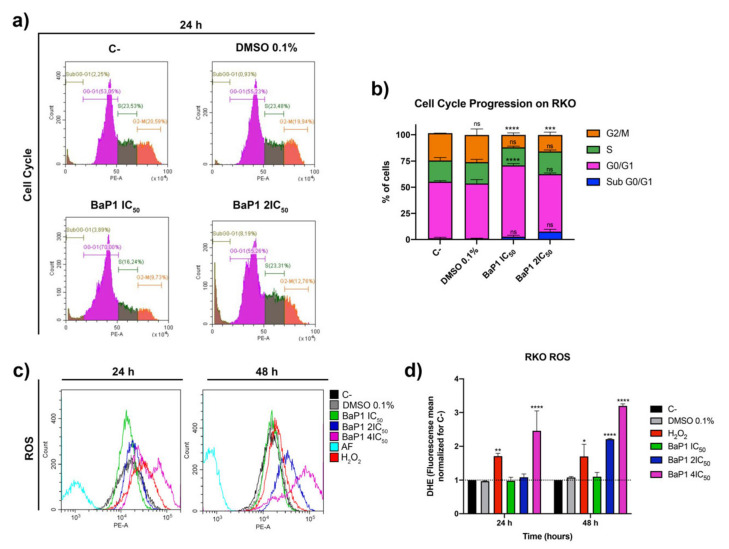
BaP1 effects on RKO cell cycle and reactive oxygen species (ROS) generation. (**a**) Representative histograms of RKO cell cycle analysis with propidium iodide after 24 h of exposure to BaP1 IC_50_ and 2 × IC_50_. Untreated cells (C-) and DMSO (0.1%) exposed cells were used as negative controls. Stage gates were established for C- and transposed for the other conditions. (**b**) Quantification of the cells in the different stages (G2/M, S, G0/G1, and Sub G0/G1). Values are means with SD (n ≥ 3). Statistical analysis was performed by two-way ANOVA. *** *p* < 0.001, **** *p* < 0.0001, ns (not significative). (**c**) Representative histograms of ROS generation analysis with dihydroethidium (DHE) after 24 and 48 h of exposure to BaP1 IC_50_, 2 × IC_50,_ and 4 × IC_50_. Untreated cells (C-) and DMSO (0.1%) exposed cells were used as negative controls and H_2_O_2_ (150 μM) as a positive control. (**d**) Quantification of the ROS levels by DHE fluorescence mean; values normalized for C-. Values are means with SD (n ≥ 3). Statistical analysis was performed by two-way ANOVA. * *p* < 0.05, ** *p* < 0.01, **** *p* < 0.0001.

Considering the high inhibitory effect and selectivity of BaP1 for RKO cells, we selected this cell line for further characterization and to investigate the biological effects of the drug. This was evaluated using proportional BaP1 concentrations with little effect on cell viability of the noncancerous cell line NCM460 and considering the biological phenotype analyzed in each assay. First, we evaluated the effect of BaP1 on cell proliferation using an optimized flow cytometry Carboxyfluorescein Diacetate Succinimidyl Ester (CFSE) staining protocol. During cell proliferation, CFSE is evenly distributed among daughter cells. This allows discrimination of successive rounds of cell division by measuring the decrease of CFSE cell fluorescence [33]. We found that CFSE cell fluorescence of the untreated cells (untreated control) decreased by more than 60% after 24 h. In contrast, CFSE cell fluorescence decreased less for the cells treated with BaP1 IC_50_ and 2 × IC_50_, by 50% and 40%, respectively (Figure 3a,b). In the following 48 h and 72 h of incubation, CFSE cell fluorescence of BaP1 treated cells decreased even less when compared to the negative control (Figure 3a,b), confirming that cell proliferation was inhibited. To determine whether the effects on cell proliferation were associated with changes in the cell cycle, we examined the effects of BaP1 IC_50_ and 2 × IC_50_ on the cell cycle by determining DNA content by using propidium iodide (PI) staining and flow cytometry. The analysis was performed after 24 h of treatment, since this time point is used as a standard for human cells. The results showed that BaP1 induced cell cycle arrest at G0/G1 stage, delaying the progression of the cell cycle predominantly for the IC_50_, with around 70% of the cells in this stage, in comparation with the 53% of the negative control, and with a low percentage of cells in G2/M stage (Figure 4a,b). The 2 × IC_50_ also affected cell cycle progression, as there was an increase in G0/G1 populations and subG0/G1 (indicative of cell apoptosis) and a significant decrease of G2/M cells (Figure 4a,b).

After proving the antiproliferative effect of BaP1, we next assessed the effect of BaP1 on cell survival by colony formation assay. We tested low doses of BaP1 (IC_50_/3, IC_50_/2, and IC_50_) and measure the ability of individual cells to grow into colonies after treatment with the compound. We found that BaP1 drastically affected cell survival, as treatment with low doses of BaP1 significantly reduced the number of colonies (Figure 3c,d). The effects were more pronounced for the IC_50_, with a reduction of more than 90% of colonies formed compared with the negative control (Figure 3c,d). In addition, colony size was smaller under the BaP1 IC_50_/2 and IC_50_ conditions (Figure 3c). Considering that cell migration is an important feature of cancer cell aggressiveness, we performed a wound-healing assay to monitor the effects of BaP1 on cell migration. BaP1 IC_50_ and 2 × IC_50_ triggered a decrease in cell migration, compared with untreated cells, after 12 h of treatment (Figure 3e,f).

Benzo[*a*]phenoxazine compounds have been reported to lead to the generation of ROS [34,35,36]. Therefore, we evaluated if BaP1 could lead to ROS generation. For this, RKO cells were treated with increasing concentrations of BaP1 (IC_50_, 2 × IC_50_ and 4 × IC_50_), and ROS were quantified by flow cytometry using dihydroethidium (DHE), a specific probe for the detection of superoxide and hydrogen peroxide. The results revealed that after 24 h of treatment with the 4 × IC_50_, a significative amount of ROS generation was induced, with a population of cells with high levels of ROS, that increased upon 48 h of treatment (Figure 4c,d). Furthermore, the 2 × IC_50_ treatment also significatively increased the levels of ROS after 48 h (Figure 4c,d). These results showed that BaP1 significantly increased the levels of ROS in RKO cells.

### 2.3. BaP1 Induces Apoptosis in RKO Cell Line

**Figure 5:**
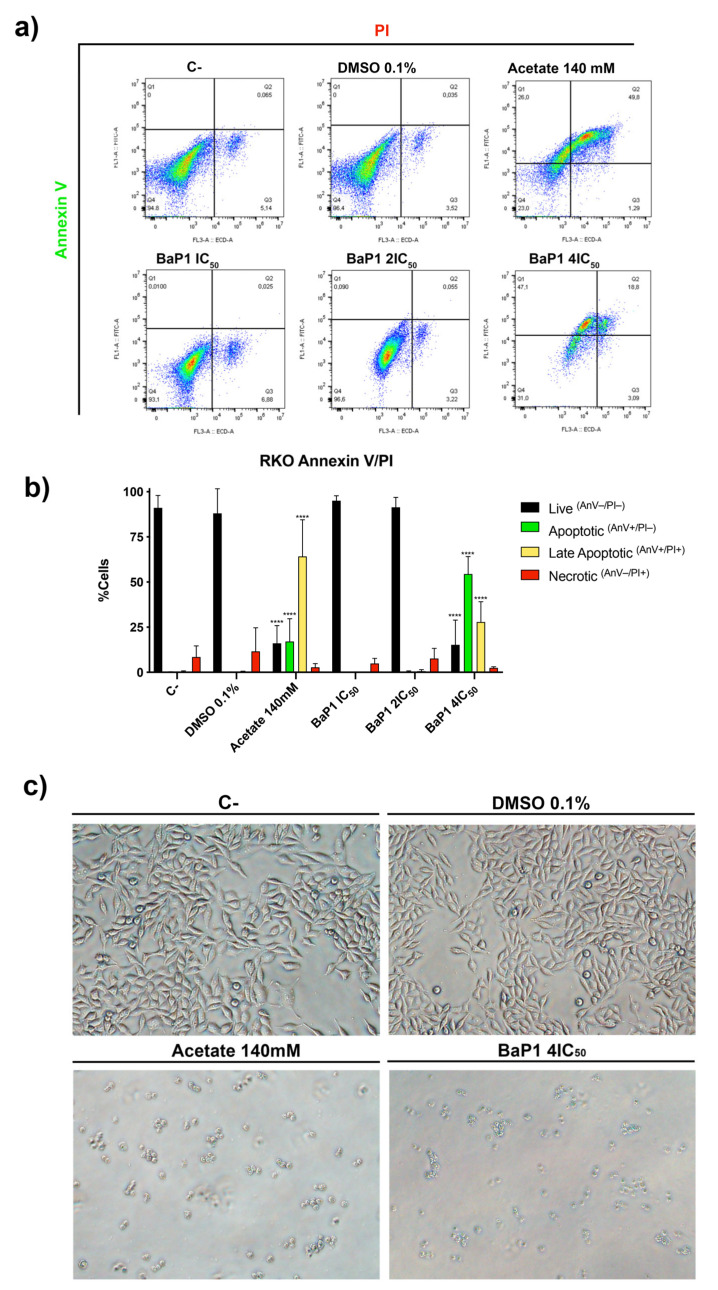
BaP1 cell death induction on RKO cell line. (**a**) Representative bi-parametric dot plots of RKO Annexin V/Propidium iodide (PI) analysis with Annexin V and PI after 48 h of exposure to increasing concentrations of BaP1 (IC_50_, 2 × IC_50,_ and 4 × IC_50_). Untreated cells (C-) and DMSO (0.1%) exposed cells were used as negative controls and acetate (140 mM) as a positive control. (**b**) Quantification of the cells in the different stages (live, apoptotic, late apoptotic, and necrotic). Values are means with SD (n ≥ 3). Statistical analysis was performed by two-way ANOVA. **** *p* < 0.0001. (**c**) Representative images of RKO apoptotic cells used in the flow cytometry Annexin V/PI analysis.

Considering the inhibitory effect of BaP1 in the RKO cell line, we next assessed if this compound could also induce significative cell death. As such, RKO cells were treated with increasing concentrations of BaP1 (IC_50_, 2 × IC_50_, and 4 × IC_50_) for 48 h and cell death was analyzed by Annexin V/PI staining through flow cytometry. We found that BaP1 induced exposure of phosphatidylserine to the outer leaflet of the plasma membrane of RKO cells in a dose-dependent manner. The number of cells stained with Annexin V (AnV^+^PI^−^ + AnV^+^PI^+^) increased from less than 1% in the negative controls (untreated control and 0.1% DMSO) to 82% after exposure to BaP1 4 × IC_50_ and to 81% when cells were exposed to 140 mM of acetate (positive control) (Figure 5a,b). Levels of necrotic cells (AnV^−^PI^+^) were very low. Furthermore, morphological observations also showed that RKO cells were affected by BaP1, as cell shrinkage was observed for the 4 × IC_50_ (Figure 5c). These results indicate that BaP1 induces apoptosis in RKO cells in a dose-dependent manner.

### 2.4. BaP1 Accumulates at the Lysosome and Induces Lysosomal Membrane Permeabilization and Cytosolic Acidification in RKO Cells

**Figure 6:**
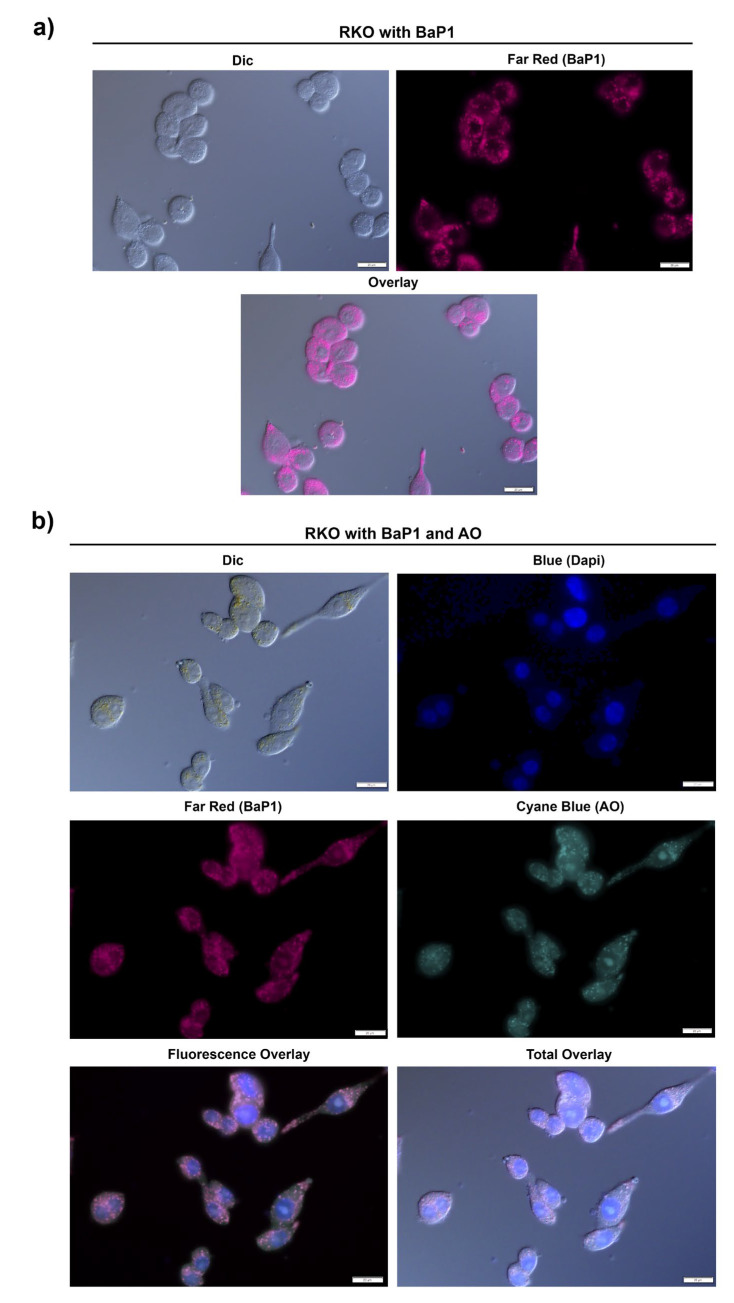

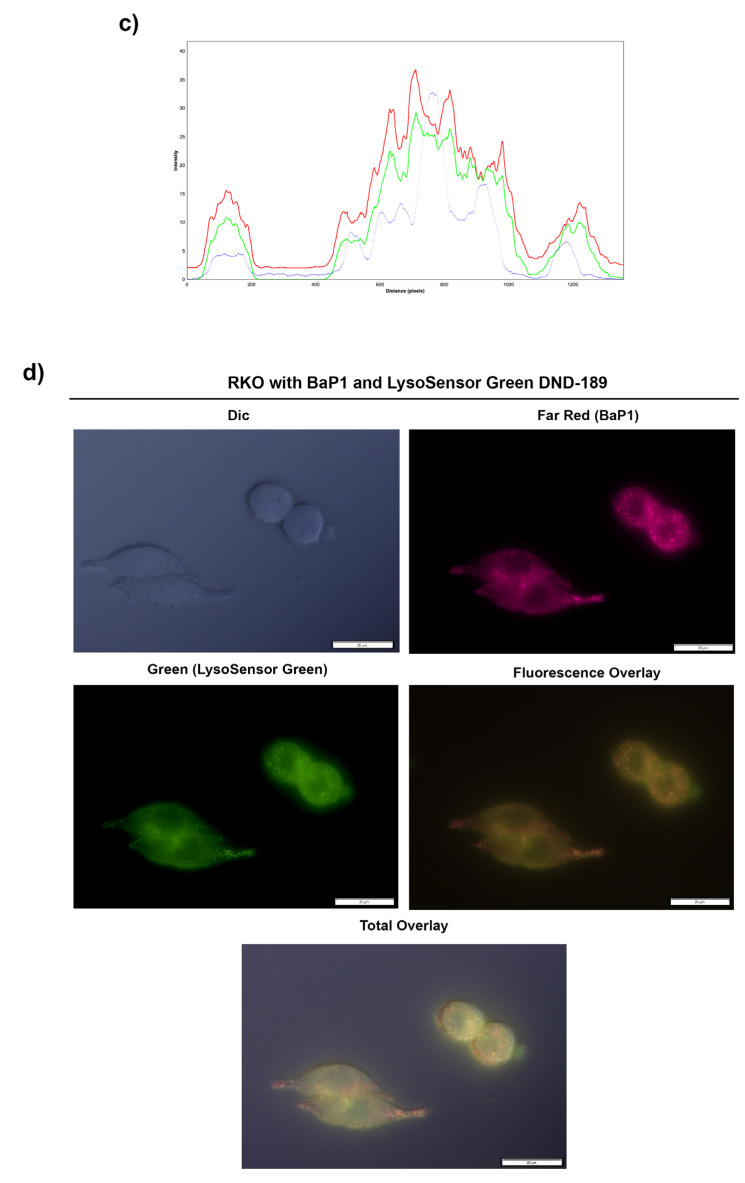
BaP1 lysosome accumulation. (**a**) Fluorescence microscopy images of RKO cells after incubation with BaP1 (0.35 μM). (**b**) Florescence microscopy images of RKO cells after incubation with BaP1 (0.35 μM), cells were stained with acridine orange (AO), represented as cyan blue (final concentration 1 μM) and co-stained with DAPI (final concentration 10 μg/mL). (**c**) RGB fluorescent emission profile of BaP1 (red line) AO (green line) and DAPI (blue line) overlay (**d**) Florescence microscopy images of RKO cells after incubation with BaP1 (0.35 μM), cells were stained with LysoSensor Green DND-189 (final concentration 2 μM).

We previously reported that BaP1 accumulates at the yeast vacuole membrane and leads to vacuolar membrane permeabilization [23]. As such, we investigated if BaP1 had an equivalent intracellular target in RKO cell line, accumulating in the equivalent organelle, the lysosome. For this, we took advantage of BaP1 intrinsic Far-Red fluorescence and co-stained cells with BaP1 and Acridine Orange (AO). AO is a weak base that when uncharged moves freely across membranes and accumulates in the acidic lysosomes where it is protonated and forms aggregates that fluoresce bright red. Using a sublethal dose we found that BaP1 fluorescence appeared in punctuated structures in RKO cells (Figure 6a), that co-localized with AO fluorescence (Figure 6b). Furthermore, this co-localization was sustained by the DIC microscopy image (where is observable AO accumulation in the lysosomes, yellow punctuated structures, which overlaps with the Far-Red fluorescence from BaP1) and by the identical florescence emission profile at between BaP1 and A.O (Figure 6c). In order to confirm this phenotype, we performed the same staining experiment co-staining cells with BaP1and LysoSensor Green DND-189, a probe that also accumulates at acidic lysosomes. The observations were identical to the staining with AO, as the green fluorescence from LysoSensor stained lysosomes co-localized with BaP1 Far-Red punctuated fluorescence (Figure 6d). These observations confirm that BaP1 accumulates in the lysosomes of RKO cells.

**Figure 7:**
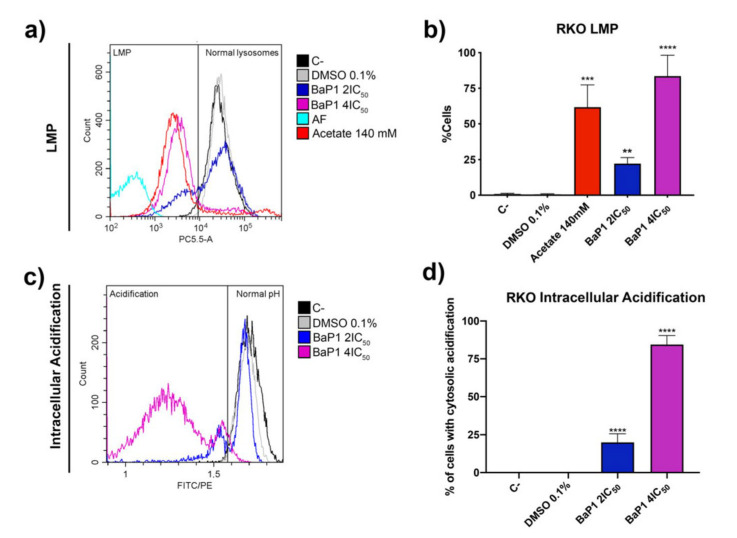
BaP1 lysosomal membrane permeabilization (LMP) and intracellular acidification induction. (**a**) Representative histograms of RKO LMP induction with AO after 48 h of exposure to BaP1 2 × IC_50_ and 4 × IC_50_. Untreated cells (C-) and DMSO (0.1%) exposed cells were used as negative controls and acetate (140 mM) as positive control. (**b**) Quantification of the cells with LMP induction. Values are means with SD (n ≥ 3). Statistical analysis was performed by one-way ANOVA. ** *p* < 0.01, *** *p* < 0.001, **** *p* < 0.0001. (**c**) Representative histograms of RKO intracellular acidification with BCECF-AM after 48 h of exposure to BaP1 2 × IC_50_ and 4 × IC_50_. Untreated cells (C-) and DMSO (0.1%) exposed cells were used as negative controls. (**d**) Quantification of the cells with intracellular acidification. Values are means with SD (n ≥ 3). Statistical analysis was performed by one-way ANOVA. **** *p* < 0.0001.

Considering these observations and the information reported for the yeast model, it seemed quite likely that the accumulation in the lysosomes ultimately results in Lysosomal Membrane Permeabilization (LMP). LMP is associated with lysosome proton release and can be measured as a decrease in the AO red fluorescence. Thus, we stained cells exposed to BaP1 4 × IC_50_ (apoptosis-inducing concentration) with AO and quantified LMP by flow cytometry, measuring the percentage of cells with loss of lysosomal AO red fluorescence. Acetate was used as positive control, as it has been demonstrated to induce LMP in CRC cells [37]. The results showed that exposure of RKO cells to BaP1 resulted in LMP occurrence in 85% of the cells (Figure 7a,b).

Intracellular acidification is reported as one of the major consequences of LMP. Considering this, we next evaluated if LMP induction by BaP1 could be associated with an intracellular acidification. We used the dual-emission ratiometric pH-sensitive probe BCECF-AM. This probe rapidly diffuses into cells were it is cleaved by esterases to its unesterified form and emits fluorescence according to the pHi (intracellular pH). Acidification is reported by the decrease of the BCECF-AM FITC/PE mean ratio. As such, the percentage of cells exhibiting intracellular acidification was estimated from the percentage of cells displaying a FITC/PE mean ratio lower than control cells (C-), defined by the gate on the histogram of Figure 7d. After 48 h of treatment, BaP1 2 × IC_50_ induced an intracellular acidification in a population of around 25% of the total cells (Figure 7c,d). Our results showed that BaP1 4 × IC_50_ had an even more severe effect, with around 90% of the cells acidified (Figure 7c,d). Interestingly is possible to observe two populations with different levels of acidification. As such, resorting to the FITC/PE mean ratios and to a standard curve obtained with cells previously buffered at different pH’s and its respective FITC/PE mean ratios we determined the pHi of the different populations. Untreated cells and DMSO (0.1%) treated cells showed a pHi of 7.8 and 7.7, respectively; 2 × IC_50_ non acidified cells presented a pHi of 7.8 and acidified cells a pHi of 6.4; and for the 4 × IC_50_ treatment, the cells with a higher percentage of acidification presented a pHi of 4.7, and the population with a lower level of acidification presented a pHi of 6.9.

### 2.5. Cathepsin D Silencing Revert BaP1 Effect in RKO Cells

**Figure 8:**
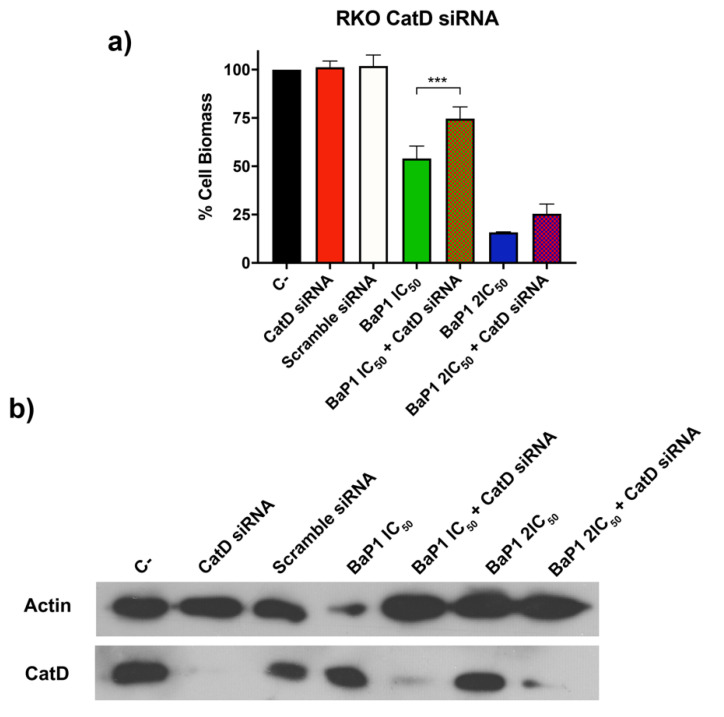
Effect of BaP1 on cell growth of Cathepsin (CatD) selected RKO cells. (**a**) Quantification of RKO cell growth assessed by SRB assay of unexposed and exposed CatD transfected cells to 48 h of BaP1 IC_50_ and 2 × IC_50_. As controls, RKO cells were not transfected (C-, BaP1 IC_50_, and 2 × IC_50_) and transfected with scrambled siRNA. Values are means with SD (n ≥ 3). Statistical analysis was performed by one-way ANOVA. *** *p* < 0.001. (**b**) Confirmation of CatD depletion by Western blot analysis of CatD expression for all the conditions. Actin was used as expression control.

The identification of lysosomes as target organelles led us to question the involvement of cathepsins as effectors in the anticancer activity of BaP1. To test this hypothesis, we examined whether depletion of Cathepsin D (CatD) could overcome the effect of BaP1. RKO cells transfected with a pool of specific small interference RNA (siRNA) targeting CatD or a scrambled non-silencing siRNA were treated with two concentrations of BaP1 IC_50_ and 2 × IC_50_ for 48 h, and differences in cell viability were analyzed. Depletion of CatD, confirmed by Western blot (Figure 8b), significantly reduced the cellular effect of BaP1 for both concentrations tested, as the CatD depleted cells were able to resist more than the cells only treated with BaP1 (Figure 8a).

### 2.6. BaP1 Reduces Tumor Growth, Angiogenesis, and Tumor Proliferation

**Figure 9:**
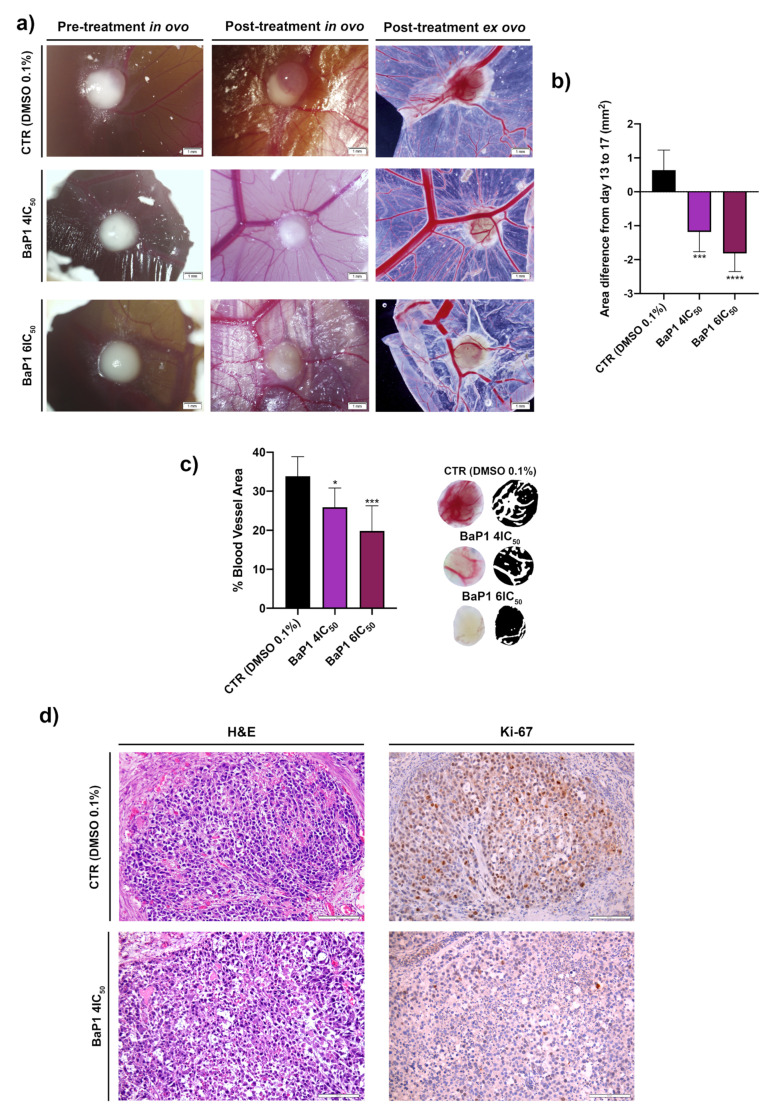
*In vivo* effects of BaP1. (**a**) Representative pictures of chicken embryo choriollantoic membrane (CAM) *in vivo* pre-treatment (day 13) and post-treatment (day 17). CAMs were exposed to DMSO (0.1%) (control group), BaP1 4 × IC_50_, and 6 × IC_50_ for 4 days. (**b**) Quantification of tumor area (difference from day 13 to day 17). (**c**) Quantification of the percentage of blood vessel area. Statistical analysis was performed by one-way ANOVA (* *p* < 0.05, *** *p* < 0.001, **** *p* < 0.0001). (**d**) Representative pictures of hematoxylin and eosin (H&E) staining and Ki-67 expression for the control group and BaP1 4 × IC_50_ treated CAMs; scale bar = 100 μm.

Our *in vitro* evaluation revealed that BaP1 has a strong anticancer activity against the RKO cell line. Therefore, we next evaluated BaP1 anticancer effect *in vivo* using the chicken embryo choriollantoic membrane (CAM) assay. RKO cells were grown in the CAM of chicken embryos for 4 days (until day 13), and then incubated with DMSO (0.1%) (control group CTR), BaP1 4 × IC_50_, and BaP1 6 × IC_50_ for 4 days (until day 17). As is possible to observe in Figure 9, the treatments with BaP1 induced a decrease in the tumor size when compared to the control group. The tumor area of the control group increased around 1 mm^2^ from the day 13 to the day 17 in contrast with the 1 and 2 mm^2^ average reduction for the 4 × IC_50_ and 6 × IC_50_ treatments (Figure 9a,b). Furthermore, the BaP1 treatments reduced the percentage of blood vessel (angiogenesis) around the tumor. The percentage of blood vessels area around the tumors of the control group was around 35% in contrast with the 25% and 20% of the 4 × IC_50_ and 6 × IC_50_ treatments, respectively. Additionally, we determined the levels of Ki-67 expression for the control group and for the 4 × IC_50_ and observed that 4 × IC_50_ of BaP1 decreased Ki-67 expression when compared to the control group, indicating that tumor cell proliferation was inhibited (Figure 9c).

## 3. Discussion

The synthesis of novel small organic molecules with great structural diversity has been one of the main drivers in the discovery of compounds for therapeutical application. In this context, in the recent years, our group has been designing and synthetizing novel benzo[*a*]phenoxazine compounds and assessing their intracellular targets and mechanisms of action in order to explore their application as therapeutical drugs [22,38,39,40,41,42].

Compounds that specifically target cancer cells while having low bystander effects in normal cells are still very limited. For the last few decades, phenoxazines have grown as molecules of great interest in anticancer therapy due to their reportedly higher effectiveness against malignant cancer cells comparatively to normal cells [32,36,43,44,45,46].

Our results show that low doses of BaP1 have a marked effect in RKO, SW480, and HTC116 CRC cell lines, but little effect in NCM460 noncancerous colon cells, suggesting a higher specificity of this compound towards cancer cells. RKO cell line displayed the highest sensitivity to BaP1, exhibiting a high selectivity index, above 9. This cell line is characterized by harboring a mutational activation of the oncogene BRAF (V600E), associated with poor prognosis for patients, related with chemoresistance, rendering patients non-responsive to established treatment regimens [47,48].

Interestingly, compounds of this family have been reported to have specific targeting abilities. This is evident in the study performed by the Gawali group who reported that two benzo[*a*]phenoxazine compounds showed specific cytotoxicity in a malignant COLO205 cell line that also harbors the activation mutation BRAF^V600E^ in comparison to a non-malignant wild-type BRAF HEK293T cell line. Their cell-based assays showed that the treatment with the two compounds resulted in a selective cell death only in BRAF^V600E^ COLO205 cells by inducing cell cycle arrest at the G0/G1 phase and caspase-mediated apoptosis [49].

One of the main problems that cancer treatment faces is that many cancer cells acquire resistance to apoptotic cell death or are less sensitive to apoptosis inducing drugs [32,50]. A more recent study has shown that molecules of this class are also capable of inducing autophagic cell death in apoptosis-resistant cancer cells. In fact, Konkimalla group reported for the first time the ability of this class of compounds to accumulate, increase granularity, and induce cell death via autophagy of an apoptosis-resistant and KRAS and p53 mutated pancreatic cancer cell line [32].

Furthermore, there are also reports of the synthesis of photosensitizers benzo[*a*]phenoxazines for photodynamic therapy in cancer. This approach is based on the focal photoactivation of photosensitizers benzo[*a*]phenoxazines that can directly act on the target tissues and elicit reactions that eventually lead to oxidative stress, resulting in direct cytotoxicity [36,51]. In fact, the first reports of the development of benzo[*a*]phenoxazines photosensitizers date back to 1991 were James W. Foley research team examined the photosensitization potency, cellular localization, and mechanism of action of some Nile Blue analogs. The authors found out that some benzo[*a*]phenoxazine derivatives harboring sulfur substitutes showed high effectiveness in photokilling tumor cells, and that their effect was associated with their accumulation at the lysosome and photodynamic destruction of these organelles through ROS membrane-degradation [52,53,54,55].

Although there are several pharmacological studies that have investigated the anticancer potential of these compounds, it should be noted, however, that there is no general description of their targets and mechanisms. Rather, it appears that the activity and behavior of the individual molecules depend entirely on the functionalizations present on the benzo[*a*]phenoxazine scaffold.

The fact that RKO cell line was more sensitive to BaP1 lead us to evaluate the biological effects of the compound in this cell line and further assess the compound intracellular targets to assess its potential as an anticancer drug. We showed that low doses of BaP1 significantly reduced RKO cell proliferation, and that this reduction was associated with blockage in cell cycle progression. Furthermore, our results reveled that BaP1 reduced cell survival and cell motility, two desirable properties for an anticancer drug.

Interestingly, when we treated RKO cells with higher doses of BaP1, 2 × IC_50_ and 4 × IC_50_, concentrations that still have a reduced inhibitory effect in the noncancerous colon cell line NCM460, we observed well-established and correlated phenotypes. For these concentration we were able to see that similarly to some compounds of this family, BaP1 lead to high levels of ROS generation and apoptotic cell death [34,35,36,40,56]. Furthermore, we observed that BaP1 accumulated on RKO cell lysosomes and caused lysosomal membrane permeabilization (LMP) and cytosolic acidification. This is in agreement with the vacuolar localization and permeabilizing effect of BaP1 in yeast cells [23], suggesting that BaP1 triggers an analogous cell death pathway both in yeast and in CRC cells, were the permeabilization of the vacuole/lysosome is the process that leads to cell death.

LMP is associated with the release and involvement of lysosomal proteases, particularly cathepsins cathepsin B, D, and L, that have been reported as executors of lysosomal-mediated cell death [57,58,59]. In the case of BaP1, our results show that the depletion of Cathepsin D reduces the BaP1 effect, a phenotype that sustains the involvement of this protease, and is consistent with our observations in the yeast model [23]. In fact, we showed that the BaP1 induced vacuolar membrane permeabilization is accompanied with release of Pep4p, yeast ortholog of cathepsin D, to the cytosol, and that Pep4p-deficient cells are more resistance to BaP1-induced cell death.

Considering the role, function, and morphological differences of lysosomes in cancer cells [11,12], it is clear that this organelle emerges as a promising therapeutical target as it shows vulnerability that can be exploited through the use of lysosome-targeting agents. In fact, induction of LMP seems to be an effective and selective way to sensitize and/or kill multidrug resistant cancer cells [5]. There are a several agents that have been reported to induce LMP, ROS being one of the main inducers [60,61]. The fact that BaP1 accumulates in the lysosome and leads to high levels of ROS generation seems to be directly correlated with BaP1 LMP induction through the damage of the lysosomal membrane. This hypothesis is consistent with the previous reports of James W. Foley on the lysosomal membrane degradation by ROS upon the photoactivation of benzo[*a*]phenoxazine derivatives [52,53,54,55]. Although photoactivation is not assessed in the case of BaP1, there are reports showing that compounds with a phenoxazine ring system can undergo enzymatic single-electron reduction by naturally occurring intracellular enzymes, resulting in the formation of radical intermediates that subsequently lead to the generation of ROS [62,63,64]. This may be the case with BaP1 and is something we intend to investigate in the future.

In our *in vivo* pre-clinical results, we observed a significant decrease in the size of the RKO tumors formed in the CAM assay, a result that validates the antitumoral activity of BaP1 against RKO cells. Furthermore, we observed that BaP1 decreased angiogenesis, characterized by a significant decrease in the vascularization formed around the tumors, as well as reduced tumor proliferation evidenced by the reduced levels of Ki67 expression in the tumors treated with the compound.

Overall, our data highlights BaP1 as a molecule with a potent and selective anticancer activity against CRC cells and as a very interesting agent to disturb and counteract the important roles of lysosome in cancer. BaP1 emerges as a promising candidate to be exploited as new anticancer targeted agent, using LMP as a therapeutic approach in CRC.

## 4. Materials and Methods

### 4.1. Human Cell Lines

We used a noncancerous cell line NCM460 and CRC-derived cell lines RKO, SW480, and HCT116. The NCM460 cell line was derived from healthy mucosal epithelium from the human colon and obtained from InCell, San Antonio, TX, USA [65]. RKO, SW480, and HCT116 are colorectal cancer-derived cell lines that harbor a mutation in BRAF^V600E^, KRAS^G12V^, and KRAS^G13D^, respectively, which were obtained from ATCC [66].

### 4.2. Growth and Culture Conditions

NCM460 and SW480 cells were grown in Roswell Park Memorial Institute (RPMI) 1640 medium with stable glutamine (Biowest, Nuaillé, France) supplemented with 1% penicillin-streptomycin (Biowest, Nuaillé, France) and 10% heat-inactivated fetal bovine serum (FBS; Gibco, Invitrogen, Waltham, Massachusetts, USA); RKO cells were grown in Dulbecco’s Modified Eagle’s Medium (DMEM) High Glucose (Biowest, Nuaillé, France) supplemented with 1% penicillin-streptomycin (Biowest, Nuaillé, France) and 10% heat-inactivated FBS (Gibco, Invitrogen, Waltham, MA, USA). HCT116 cells were grown in McCoy’s 5A Medium (Biowest, Nuaillé, France) supplemented with 1% penicillin-streptomycin (Biowest) and 10% heat-inactivated FBS (Gibco, Invitrogen, Nuaillé, France). All cell lines were plated onto 25 cm^3^ tissue culture flasks, maintained in a humidified incubator with 5% CO_2_ at 37 °C.

### 4.3. SRB Assay

The effect of the BaP1 on cell viability was determined by the sulforhodamine B (SRB) assay in RKO, SW480, HCT116, and NCM460 cell lines. Cells were seeded with a density of 4 × 10^4^ (RKO), 1.75 × 10^5^ (SW480), 5 × 10^4^ (HCT116), and 3 × 10^5^ (NCM460) cells/well and allowed to adhere for 24 h at 37 °C with 5% CO_2_. On the next day, increasing concentrations (1 μM, 2.5 μM, 5 μM, and 7.5 μM) of the compound were added to the respective wells and the plates were incubated for 48 h. For the SW480 cell line, a 6 μM concentration of BaP1 was also tested, as well as 0.5 μM of BaP1 for the HCT116 cell line. Besides the untreated control (C-), with cells and growth medium only, the highest concentration of the solvent where the compound was dissolved (DMSO 0.1%) was also used as vehicle. The SRB assay was performed according to [37].

### 4.4. Assessment of Cell Proliferation with CFSE

Carboxyfluorescein Diacetate Succinimidyl Ester (CFSE) labeling was performed for the RKO cell line. Cells were labeled before seeding. Briefly, cells were collected, washed with PBS, and incubated with CFSE (5 μM final concentration) for 15 min at 37 °C in a water bath. Afterward, cells were rinsed with medium, resuspended in the correct amount of medium, and seeded in 6-well plates at 1 × 10^5^ cells/mL. After adhering for 24 h, cells were treated with fresh complete medium (untreated control) or DMSO (0.1%) as negative controls, as well as with BaP1 IC_50_ and 2 × IC_50_. Cells were harvested at different time points 0, 24, 48, and 72 h after treatment, and the CFSE median fluorescence intensity was analyzed by flow cytometry using the FITC-A channel. At the moment of the seeding, a sample from the labeled cell suspension was also collected and analyzed to ensure correct cell staining. All median values were normalized to the time point 0 h.

### 4.5. Colony Formation Assay

A colony formation assay was performed for the RKO cell line; 600 cells/mL were seeded in 6-well plates. After adhering for 24 h, cells were treated with fresh complete medium (untreated control) or DMSO (0.1%) as negative controls, as well as with BaP1 IC_50_, IC_50_/2, and IC_50_/3. After 48 h of treatment, the medium containing the compounds was removed, cells were washed twice with PBS, and then fresh medium was added. Cells were then allowed to grow for 10–14 days (the medium was changed every 3 days). The colonies were washed with PBS and fixed for 30 min with 6% glutaraldehyde and 0.5% crystal violet. The number of colonies was counted using ImageJ Software, and the percentage of colonies was normalized for the untreated control.

### 4.6. Wound Healing Assay

RKO cells were seeded in 6-well plates at a density of 5 × 10^5^ cells/mL. After adhering for 24 h, the wound was performed by scraping the cell layer in a straight line using a 1 mm pipette tip. At this time, the wound areas were photographed (time 0), and the cells were treated with fresh complete medium (untreated control) or DMSO (0.1%) as negative controls, as well as with BaP1 IC_50_ and 2 × IC_50_. The wound areas were photographed at 4, 8, and 12 h. The relative migration distances were then analyzed using Image J Software.

### 4.7. Cell Cycle Analysis

The alterations on the cell cycle were evaluated by the measurement of the DNA content. 1 × 10^5^ cells/mL of RKO cell line were seeded in 6-well plates. After adhering for 24 h, cells were treated with fresh complete medium (untreated control) or DMSO (0.1%) as negative controls, as well as with BaP1 IC_50_ and 2 × IC_50_. After 24 and 48 h of treatment, cells were collected, resuspended in 500 μL PBS, and incubated on ice for 15 min. After this, 1.5 mL of 96% (*v*/*v*) cold ethanol was added, and the cells were incubated for 15 min on ice. Cells were then washed, resuspended in 500 μL of PBS, and incubated with 50 μL of RNase A solution [200 μg/mL in sodium citrate 1% (*w*/*v*)] at 37 °C for 15 min. After incubation, 50 μL propidium iodide (PI) staining solution [0.5 mg/mL in sodium citrate 1% (*w*/*v*)] was added and the cells were mixed in a vortex and were incubated at room temperature for 30 min in the dark. PI mean fluorescence was analyzed by flow cytometry using the PE-A channel.

### 4.8. ROS Detection

The changes of cellular ROS levels were measured using dihydroethidium (DHE) by flow cytometry. Using the RKO cell line, 1 × 10^5^ cells/mL were seeded in 12-well plates. After adhering for 24 h, cells were treated with fresh complete medium (untreated control) or DMSO (0.1%) as negative controls, 150 μM of H_2_O_2_ (positive control), as well as with BaP1 IC_50_ and 2 × IC_50_ and 4 × IC_50_. Cells were collected after 24 and 48 h of treatment, washed with PBS, and stained with 0.5 μM DHE for 30 min at 37 °C in the dark. DHE mean fluorescence intensity was analyzed by flow cytometry using the PE-A channel.

### 4.9. Annexin V/PI Staining Assay

Using the RKO cell line, 1 × 10^5^ cells/mL were seeded in 6-well plates. After adhering for 24 h, cells were exposed to increasing concentrations of BaP1 IC_50_, 2 × IC_50_ and 4 × IC_50_ for 48 h. Cells were incubated with fresh complete medium (untreated control) or DMSO (0.1%) as negative controls and with 140 mM of acetate as positive control. After 48 h, both floating and attached cells were collected and washed in PBS. Cells were resuspended in 100 mL of binding buffer and incubated with 5 μL of Annexin V(AnV)-FITC (Detection Kit–ab14085) and 5 μL of Propidium Iodide (PI) (50 μg/mL) for 15 min in the dark. To measure autofluorescence, cells were incubated without or with both probes separately. Samples were analyzed by flow cytometry, mono-parametric detection of PI fluorescence was performed using the ECD-A channel and mono-parametric detection of Annexin V fluorescence was performed using the FITC-A channel.

### 4.10. BaP1 Lysosome Accumulation and Lisosomal Membrane Permeablization Assessment (LMP)

Lysosome staining was achieved using Acridine Orange (AO) and LysoSensor Green DND-189. RKO cells where plated on microscopy slides with a density of 1.5 × 10^5^ (RKO) and allowed to adhere for 24 h at 37 °C with 5% CO_2_. On the next day, cells where exposed to 0.35 μM of BaP1 for 3 h. Cells were washed and resuspended in PBS, stained with 1 μM of AO for 15 min at 37 °C or with 2 μM of LysoSensor Green for 30 min at 37 °C. AO-stained cells were co-stained with DAPI (final concentration 10 μg/mL). The samples were analyzed on an Olympus BX6F2 microscope, with appropriate filter cubes: U-FDICT (differential interference contrast), TLV-U-FF-FITC (green), U-FYW (far-red), U-FGNA (red) and U-FUNA (blue), with 40x and 60x oil immersion objectives. AO fluorescence was represented as cyan blue, for co-localization purposes.

LMP was accessed by analysis of AO staining by flow cytometry. Using the RKO cell line, 1 × 10^5^ cells/mL were seeded in 6-well plates and allowed to adhere for 24 h. Cells were incubated with fresh complete medium (untreated control) or DMSO (0.1%) as negative controls and with 140 mM acetate (positive control) as well as with BaP1 4 × IC_50_. After 48 h, both floating and attached cells were collected, washed with PBS, and resuspended in PBS. Cells were then incubated with 1 μM AO (or without AO to measure autofluorescence) for 15 min at 37 °C. Samples were analyzed by flow cytometry; AO fluorescence detection was performed using the PC5.5 channel.

### 4.11. RNA Interference-Mediated Inhibition of Cathepsin D

RKO cells were seeded in 24-well plates at a density of 4 × 10^4^ cells per well. After 24 h, cells were transfected with 15 nM on-target plus SMART pool siRNA against Cathepsin D (CatD) (A-003649-16; Thermo Fisher Scientific, Lafayette, CO, USA). Transfection performance was monitored using a validated Silencer Select Negative Control (scrambled siRNA control, no. 4390843; Life Technologies, Carlsbad, CA, USA). Transfection was performed with 9 μL of HiPerFect transfection reagent (Qiagen, Hilden, Germany). After 15 h, the transfection mixture was removed, and cells were left untreated (untreated control) or treated with BaP1 IC_50_ and 2 × IC_50_ and incubated for a further 48 h in fresh medium. After 48 h, the effects of the BaP1 on cell viability were determined by SRB (as described above). CatD levels were monitored by Western blotting that was performed according to [67].

### 4.12. Intracellular pH Measurement

Measurements of the intracellular pH (pHi) were performed with the pH-sensitive probe BCECF-AM. Using the RKO cell line, 1 × 10^5^ cells/mL were seeded in 6-well plates. After adhering for 24 h, cells were exposed to increasing concentrations of BaP1 2 × IC_50_, and 4 × IC_50_ for 48 h. Cells were incubated with fresh complete medium (untreated control) or DMSO (0.1%) as negative controls. After 48 h, both floating and attached cells were collected, washed, and resuspended in Hank’s balanced salt solution (HBSS). After, cells were stained with 1 μM of BCECF-AM for 30 min at 37 °C. Samples were analyzed by flow cytometry. BCECF-AM fluorescence mean detection was performed using the FITC-A and PE-A channels. The percentage of cells exhibiting intracellular acidification was estimated from the percentage of cells displaying a FITC-A/PE-A ratio lower than control cells. The pHi was quantified using a standard curve. For this, 2.5 × 10^5^ cells/mL were incubated with 1 μM of BCECF-AM for 30 min at 37 °C. Stained cells were washed with ice cold HBSS, collected, placed on ice, and resuspended in six different PBS solutions, at pH 5.5, 6.5, 7, 7.5, and 8, supplemented with 10 μM of Nigericin. Samples were then analyzed by flow cytometry. The FITC-A/PE-A ratio from the pH series was used to create the standard curve and a linear equation. The ratio value of the cells with cytosolic acidification was used to determine the pHi for the different treatments.

### 4.13. In Vivo Chick Chorioallantoic Membrane (CAM) Assay

Fertilized chicken eggs were incubated at 37 °C. On day 3 of embryo development, a window was made into the eggshell, sealed with BTK tape and the eggs were returned to the incubator. On day 9 of embryo development, an RKO cell line suspension (2 × 10^6^ cells in 10 μL of Matrigel (Corning: 354230)) was placed inside the eggs to allow the formation of a 3D tumor. On day 13 of development, the tumors were treated with 20 μL of DMSO (0.1%) (control group), 20 μL of BaP1 4 × IC_50_, and 20 μL of BaP1 6 × IC_50_. After 96 h of treatment (day 17 of development), the chicken embryos were sacrificed by placing them at −80 °C for 10 min. Digital images of the tumors were taken on days 13 and 17 of development in a stereomicroscope (Olympus S2 × 16), using a digital camera (OlympusDP71). At the selected time-points, the “*in ovo*” tumor area was measured using ImageJ software. The results were expressed as the area difference between day 13 and 17. For blood vessel analysis, “*ex ovo*” images were analyzed in Fiji software using the “Vessel analysis” plugin, and the results expressed as percentage of blood vessel area. The tumors were fixed in 4% paraformaldehyde at room temperature and included in paraffin for further analysis.

### 4.14. Hematoxylin and Eosin Staining

Histological slides with 4 μm-thick tissue sections were subjected to Hematoxylin and eosin (H&E) staining. Briefly, sections were deparaffinized with xylene, rehydrated in ethanol, and stained with hematoxylin and eosin. Afterwards, sections were dehydrated and mounted with resinous mounting medium. The stained slides were evaluated and photographed using the Olympus BX6F microscope with a 40× immersion objective.

### 4.15. Immunohistochemistry

Histological slides with 4 μm-thick tissue sections were subjected to immunohistochemistry using a polymer system (UltraVision ONE Detection System: HRP Polymer Lab Vision Corporation, Fremont, CA, USA), as previously described [68]. Briefly, deparaffinized and rehydrated slides were incubated with 10 mM citrate buffer (pH 6.0) for 15 min in a microwave at 600 W for antigen retrieval. Then, the sections were incubated overnight at room temperature with a primary anti-Ki-67 antibody (Biolegend ref: 350502, dilution 1:100). The immune reaction was visualized using 3,3′-Diamonobenzidine (DAB Substrate Kit abcam (ab64238)) as chromogen, and tumor tissue sections were counterstained with hematoxylin. The stained slides were evaluated and photographed using the Olympus BX6F microscope with a 40× immersion objective.

### 4.16. Flow Cytometry and Fluorescence Microscopy

Flow cytometry samples were analyzed in flow cytometer Cytoflex System B4-R2-V0 (Beckman Coulter), equipped with 488 nm solid state laser (50 mW), FS, SS, FITC (525/40 BP), PE (585/42), ECD (610/20 BP), and PC5.5 (690/50 BP) channels. Twenty thousand cells were analyzed per sample at low flow rate. Flow cytometry analyses was performed with FlowJo^®^ 7.6 and CytExpert Data software.

The microscopy samples were analyzed on an Olympus BX6F2 microscope with appropriate filter cubes: U-FDICT (differential interference contrast), U-FYW (far-red), U-FGNA (red), and U-FUNA (blue), with a 60× oil immersion objective. Images were processed using Olympus ImageLS software.

### 4.17. Statistical Analysis

Results were obtained from at least three independent experiments and expressed as means ± SD. Results were analyzed by one-way or two-way ANOVA with Dunnett’s post-test. *p*-values lower than 0.05 were considered statistically significant. Statistical analyses were performed using GraphPad Prism version 8.2.1 for macOS.

## 5. Conclusions

In summary, in this study we explored the anticancer potential of a new benzo[*a*]phenoxazine compound - BaP1 for CRC treatment and evaluated the underlaying mechanisms associated with its activity. We found that BaP1 is selective for CRC cells, reduces cell proliferation, cell survival, and cell migration. In addition, we found that BaP1 accumulates on the lysosome of CRC cells and leads to ROS accumulation, lysosomal membrane permeabilization, cytosolic acidification and apoptotic cell death. Furthermore, our in vivo results, using CAM assay, sustained the anticancer activity of BaP1, as it was found that BaP1 inhibits tumor growth, angiogenesis and tumor proliferation, relevant hallmarks of cancer. Therefore, our study highlights BaP1 as an interesting agent to disturb and counteract the important roles of lysosomes in cancer and suggests BaP1 as a promising candidate to be exploited as new anticancer lysosomal-targeted agent, which uses lysosome membrane permeabilization (LMP) as a therapeutic approach in CRC.

## Data Availability

The data presented in this study are available on request from the corresponding author.
